# Spike Correlations in a Songbird Agree with a Simple Markov Population Model

**DOI:** 10.1371/journal.pcbi.0030249

**Published:** 2007-12-21

**Authors:** Andrea P Weber, Richard H. R Hahnloser

**Affiliations:** Institute of Neuroinformatics UZH/ETH Zurich, Zurich, Switzerland; University College London, United Kingdom

## Abstract

The relationships between neural activity at the single-cell and the population levels are of central importance for understanding neural codes. In many sensory systems, collective behaviors in large cell groups can be described by pairwise spike correlations. Here, we test whether in a highly specialized premotor system of songbirds, pairwise spike correlations themselves can be seen as a simple corollary of an underlying random process. We test hypotheses on connectivity and network dynamics in the motor pathway of zebra finches using a high-level population model that is independent of detailed single-neuron properties. We assume that neural population activity evolves along a finite set of states during singing, and that during sleep population activity randomly switches back and forth between song states and a single resting state. Individual spike trains are generated by associating with each of the population states a particular firing mode, such as bursting or tonic firing. With an overall modification of one or two simple control parameters, the Markov model is able to reproduce observed firing statistics and spike correlations in different neuron types and behavioral states. Our results suggest that song- and sleep-related firing patterns are identical on short time scales and result from random sampling of a unique underlying theme. The efficiency of our population model may apply also to other neural systems in which population hypotheses can be tested on recordings from small neuron groups.

## Author Summary

To deal with the vast complexity of the brain and its many degrees of freedom, many reductionist methods have been designed that can be used to simplify neural interactions to just a few key underlying macroscopic variables. Despite these theoretical advances, even today relatively few population models have been subjected to stringent experimental tests. We explore whether second-order spike correlations measured in songbirds can be explained by single-neuron statistics and population dynamics, both reflecting hypotheses on network connectivity. We formulate a Markov population model with essentially two degrees of freedom and associated with different behavioral states of birds such as waking, singing, or sleeping. Excellent agreement between spike-train data and model is achieved, given a few connectivity assumptions that strengthen the view of a hierarchical organization of songbird motor networks. This work is an important demonstration that a broad range of neural activity patterns can be compatible at the population level with few underlying degrees of freedom.

## Introduction

Spontaneous neural activity in the absence of sensory stimulation (e.g., during sleep) often exhibits stereotyped sequences that can resemble sensory or motor sequences [1–5]. A central question pertaining to such observations is the extent to which spike sequences in single neurons reflect sequential behaviors across larger populations. Sometimes there is strong correspondence, and the spike patterns in single neurons can be precisely predicted from a coarse population readout [6]. However, it is largely unexplored whether population-conditional models of spike trains can go beyond single-neuron statistics and also explain pairwise spike correlations.

Pairwise spike correlations can signal important information beyond that of firing rates [7,8], and in some sensory systems no higher-order interactions seem to exist beyond that of cell pairs [9]. Spike correlations can be interpreted as evidence either of direct synaptic interactions or of common synaptic inputs. To illustrate the relationship between spike correlations and population models, let us consider neurons that display some regular subthreshold oscillations and occasionally fire a spike at the peaks of oscillation cycles. From single-unit data, we cannot infer the activity distribution across the population. However, given pairwise spiking data, we can estimate the number of population states from the conditional probability that a cell spikes given that a spike in another cell occurs (which is a measure of spike correlation). For example, if the conditional spike probability (CSP) averaged over cell pairs is one, then all cells must be linked to the same population state, and fire with unit probability when that state is visited. If, on the other hand, CSPs average to 0.2, then the cells can be distributed among at most five equiprobable states. For example, neurons could each be randomly linked to one of five states and fire with unit probability when that state is visited; or they could all be linked to the same state and fire with probability 0.2 when that state is visited. Which of these cases applies depends on the spread of CSPs: in the one-state case, all CSPs would be narrowly distributed around 0.2, and in the five-state case, CSPs would be bimodally distributed around zero and one (and average to 0.2). The point of this hypothetical example is to illustrate that population-conditional models are constrained by spike correlations, and therefore such models must be tested on experimental data.

**Figure 1:**
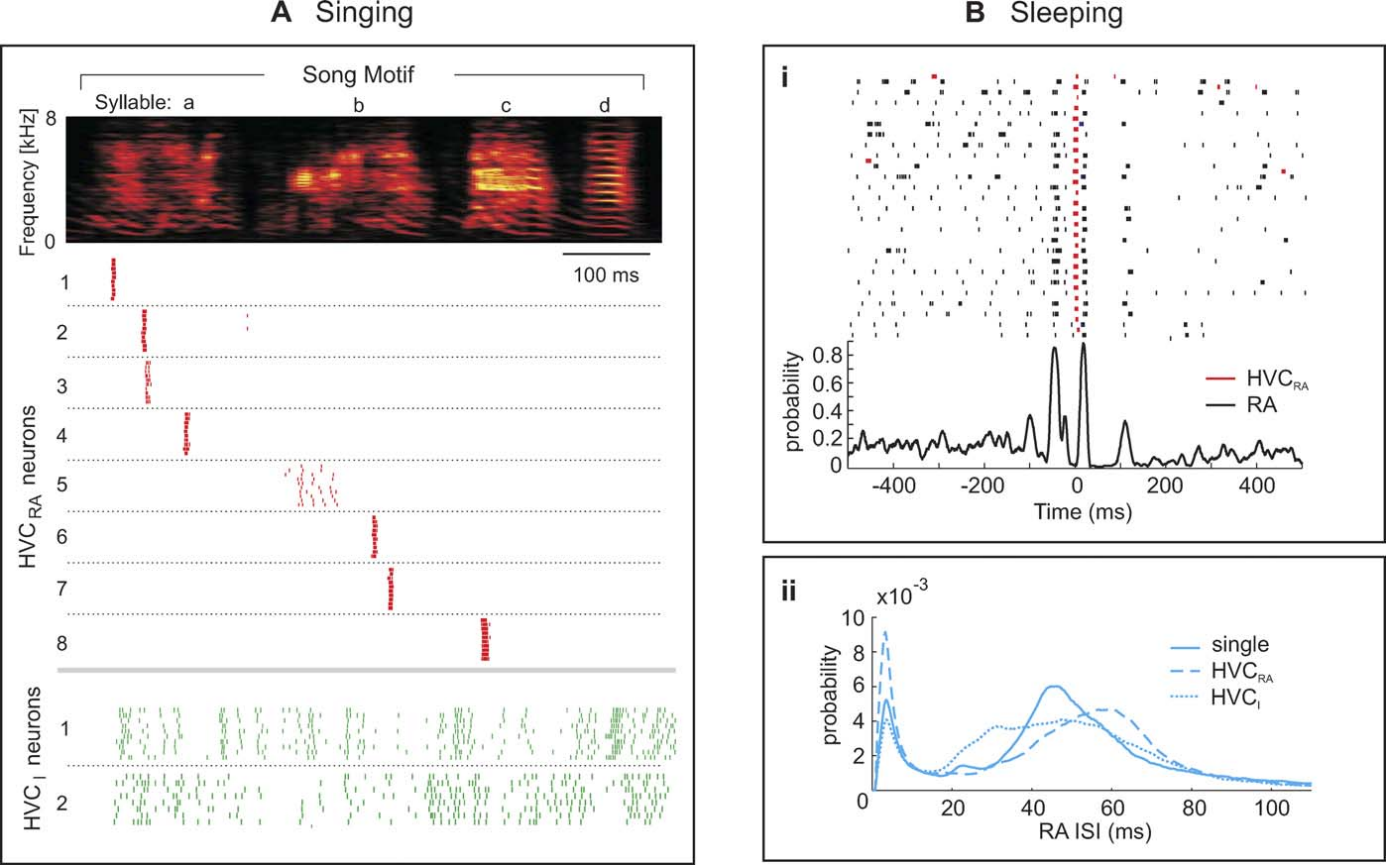
Song and Sleep-Related Firing in HVC and RA Neurons of Zebra Finches (A) During the production of a song motif (sound spectrogram on top), RA-projecting HVC neurons (HVC_RA_ neurons) produce at most one stereotyped spike burst (red rasters). HVC interneurons (HVC_I_ neurons) produce dense and less-stereotyped spike patterns (green rasters). A more elaborate version of this figure was originally published in [1]. (B) Sleep-related firing in HVC_RA_ and RA neurons. (i) Top: spike-raster plot of a simultaneously recorded HVC_RA_–RA pair during sleep. RA spikes (black rasters) have been time aligned to HVC_RA_ bursts (red rasters). (i) Bottom: CSP function of the same neuron pair. Also known as the cross-intensity function, the CSP function is an estimate of the conditional RA spiking probability as a function of the time lag to HVC_RA_ spikes (see Methods). (ii) ISI pdfs of RA neurons vary from one dataset to another. ISI pdfs have been averaged either over 29 RA neurons recorded in isolation (full line), or over 26 RA neurons recorded simultaneously with HVC_RA_ neurons (dashed line), or over 50 RA neurons recorded simultaneously with HVC_I_ neurons (dotted line).

In the robust nucleus of the arcopallium (RA) and the high vocal center (HVC) of zebra finches, neurons exhibit precise and stereotyped high-frequency bursts during singing. The number of bursts produced per song motif varies strongly between neuron types, from about one burst in RA-projecting HVC neurons (HVC_RA_ neurons), to about 12 bursts in RA projection neurons, and up to more than 20 bursts in HVC interneurons (HVC_I_ neurons) (Figure 1A) [1,10,11]. In awake, non-singing birds, RA and HVC neurons do not burst and are either silent or in a mode of tonic firing [12,13]. And during sleep they display incessant switching between bursting and tonic firing modes; in RA neurons, the sleep-related burst patterns can be highly similar to song-related patterns [4], and often the patterns are time-locked to bursts in simultaneously recorded RA-projecting HVC neurons (Figure 1Bi) [12].

Inspired by these data, we study a simple Markov model of neural populations that is based on a chain network of synaptic connections among HVC_RA_ neurons [14,15]. Model spike trains depend on the sequence of population states and are otherwise independent of each other. Formally, state–space models allow for the a priori estimation of the state dynamics from given spike data [16–19]. However, because here we assume knowledge of the state–space topology (i.e., a chain-like network among HVC_RA_ neurons), we are faced with the simpler problem of estimating the transition probabilities associated with the chain.

We explore to what fraction sleep-related bursts in HVC and RA constitute replay of premotor bursts. We compare our simulations to sets of song- and sleep-related spike data in different HVC and RA neuron types [1,10–12]. These datasets are affected by a nonnegligible variability, as exemplified by averages of sleep-related interspike interval (ISI) distributions in RA neurons (Figure 1Bii). This variability entails model parameters needing to be individually adjusted for each dataset. Our main finding is that the diversity of the data across sets and across behavioral states (waking, singing, and sleeping) can be essentially ascribed to two macroscopic transition probabilities; these set the likelihood that population activity either evolves along the chain of motor states imprinted in the HVC_RA_ network, or flips back and forth between motor states and a single resting state. Our results strengthen the view that synaptic networks are organized to support well-defined and highly constrained population behaviors.

## Results

### Model

**Figure 2:**
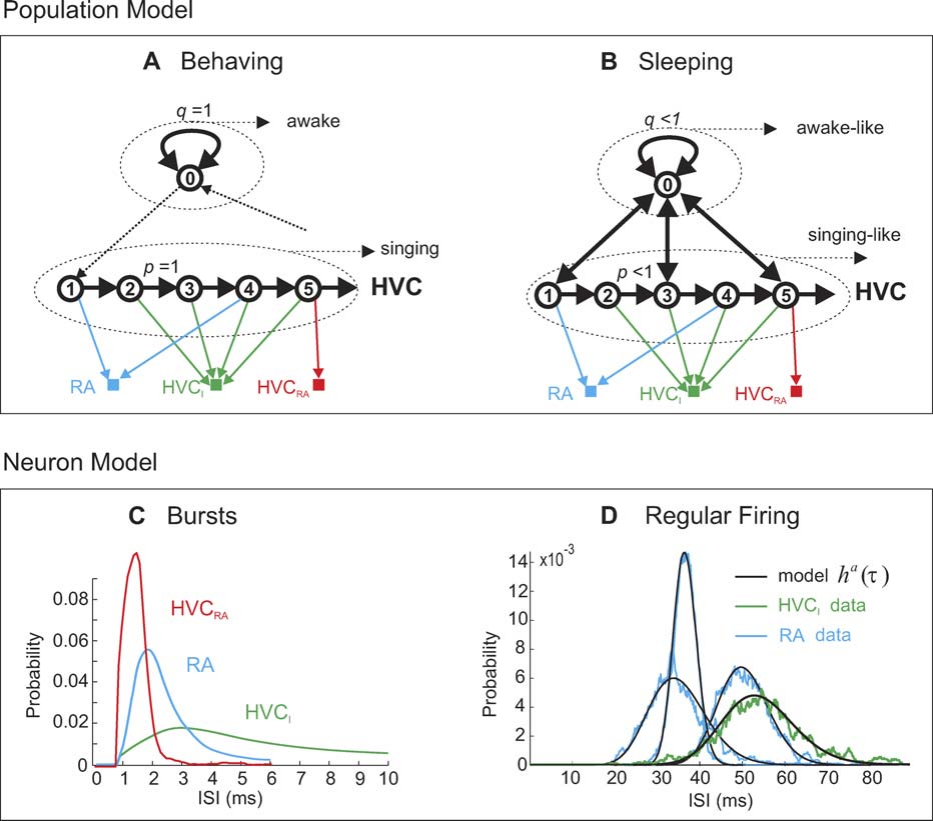
Markov Model of HVC Activity during Behavior and Sleep (A) When birds are awake, but not singing, HVC activity persists in a ground state (state 0) with probability *q* = 1. When birds sing, groups of HVC_RA_ neurons (numbered circles) are sequentially activated with probability *p* = 1 (the dashed arrows indicate song onset and offset). A single HVC_RA_ neuron (red square) is linked with exactly one HVC_RA_ group, and single RA and HVC_I_ neurons (blue and green squares) are linked with random subsets of *L*_R_ and *L*_I_ groups, respectively. (B) During sleep, HVC_RA_ groups are sequentially activated with probability *p* < 1; with probability 1 − *p*, HVC activity transits into the ground state. There, it persists with probability *q* < 1; with probability 1 − *q*, it transits back into a song state. (C) Bursts in different neuron types are modeled by the first few milliseconds of averaged song-related ISI pdfs *p^b^*(τ). (D) Tonic firing in RA and HVC_I_ neurons is modeled by gamma functions *p^a^*(τ) (black curves). The diversity of waking-related ISI pdfs in these neurons is illustrated by the blue and green curves, each representing a different neuron.

In our model, HVC population activity is a random variable that evolves in roughly 5 ms steps and is either in the ground state, or in one of 100 song states. The number of song states is chosen such that a total song-motif duration of 500 ms results [20]. Each of the song states corresponds to activation of a virtual group of 50–150 RA-projecting HVC neurons (referred to as HVC_RA_ neuron groups, or simply HVC_RA_ groups). During singing, HVC_RA_ groups are activated sequentially with probability *p* = 1 (Figure 2A). When birds are awake, but not singing, HVC activity remains in the ground state (state 0) with probability *q* = 1. During sleep, HVC_RA_ groups are also sequentially activated, but with reduced probability *p* < 1, and, the persistence probability in the ground state is also reduced to *q* < 1 (Figure 2B). By construction, neurons remain for exponentially distributed times in song and ground states during sleep, in agreement with recent estimates [17].

Given a sequence of states that describes HVC population activity, we generated spike trains in individual neurons by random sampling of model ISI probability density functions (pdfs). We assumed that HVC_RA_ neurons are each randomly linked to exactly one HVC_RA_ group and fired a burst only when that group was activated; otherwise they remained silent. HVC_I_ and RA neurons were randomly linked to more than one HVC_RA_ group and fired several bursts per song motif. For each neuron type, burst ISI pdfs were fixed and were simply derived from measurements (Figure 2C). Interestingly, in all neuron types, sleep-related bursts have lower firing rates than song-related bursts (see Figure S1). To accommodate this fact, model pdfs had to be slowed down during sleep (see Methods for details). Finally, when HVC activity was in the ground state, HVC_RA_ neurons remained silent, whereas RA and HVC_I_ neurons generated ISIs sampled from gamma functions (Figure 2D). Because waking-related RA and HVC_I_ firing rates are very diverse [12], the means of gamma functions were kept as free parameters together with *p* and *q*. Descriptions and derivations of model parameters are summarized in Table 1.

**Table 1:**
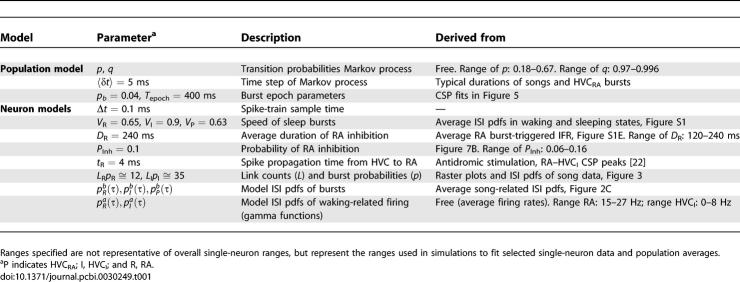
Model Parameters and Their Derivation

### Fits to Song-Related and Sleep-Related Data

**Figure 3:**
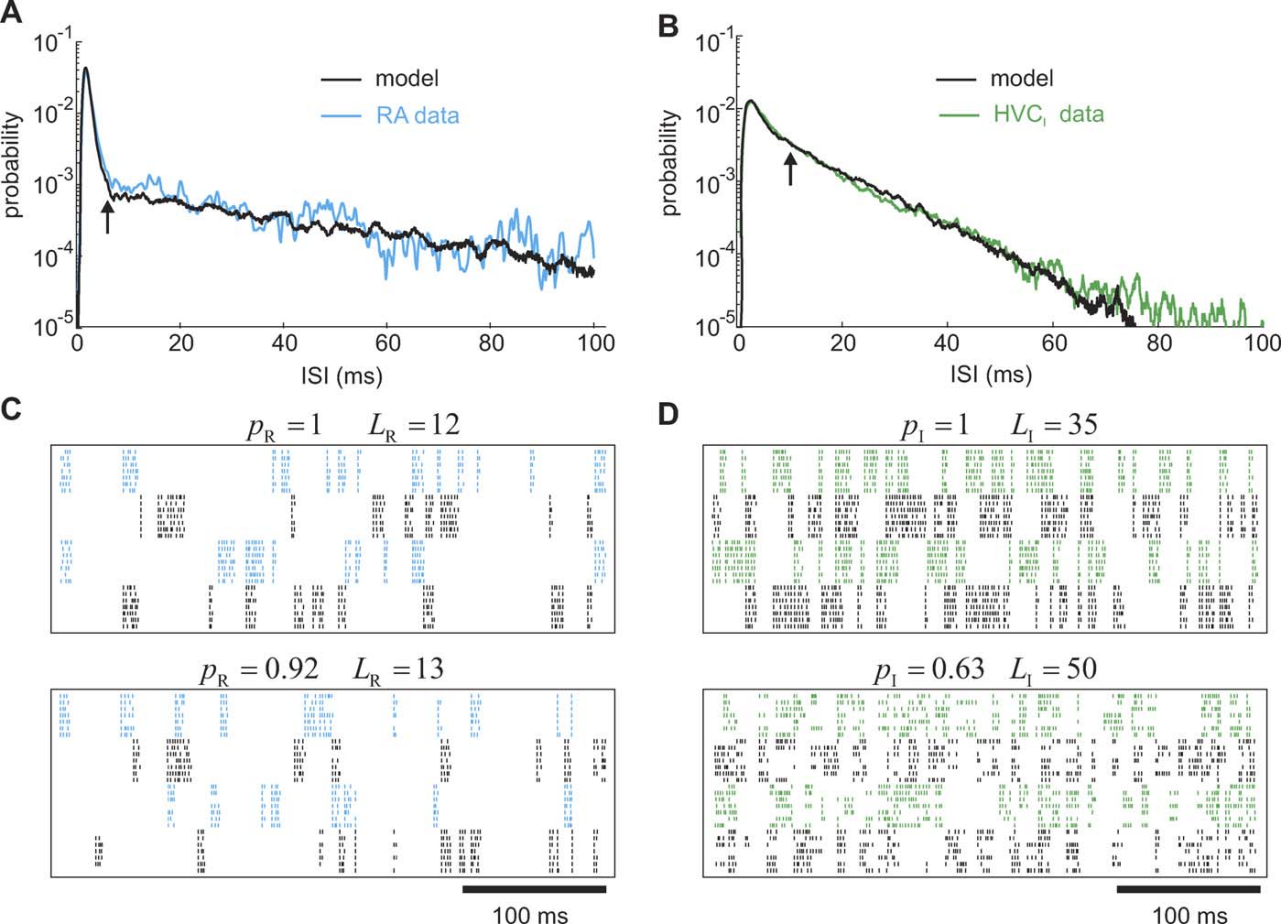
Song-Related ISI pdfs of RA and HVC_I_ Neurons (A,B) Model-based fits of averaged ISI pdfs in RA and HVC_I_ neurons during singing. The arrows delimit the ISI range of the burst models in Figure 2C, i.e., 6 ms and 10 ms, respectively. The RA-neuron data (A) were taken from [10], and the HVC_I_ data (B) were provided courtesy of A. Kozhevnikov. *L*_R_ = 12, and *L_I_* = 35. (C,D) Raster plots of song-related spike trains in four RA and four HVC_I_ model neurons for two different values of link counts *L*_R/I_ and burst probabilities *p*_R/I_. Spikes are represented as tick marks and drawn in alternating colors for different neurons.

We found that song-related ISI pdfs beyond the burst scale could be well fit over the entire ISI range (up to 100 ms) by randomly linking RA neurons to *L*_R_ = 12 HVC_RA_ groups and HVC_I_ neurons to *L*_I_ = 35 groups (Figure 3A and 3B). Note that the larger the link counts *L*_R_ and *L*_I_, the steeper were the corresponding exponential tails of the pdfs. However, to also account for the considerable lack of stereotypy mainly in raster plots of HVC_I_ neurons [11], we had to trade off high link counts against reduced burst probabilities (the probability that a neuron bursts when an HVC_RA_ group to which it is linked is activated). Note that a less than unit burst probability can be interpreted as a reduction in neural responsiveness to excitatory synaptic drive, or as increased inhibition. We obtained good results with burst probabilities in RA neurons of *p*_R_ = 0.92 (*L*_R_ = 13) and in HVC_I_ neurons *p*_I_ = 0.63 (*L*_I_ = 50) (Figure 3C and 3D). Note that first-order statistics impose the following constraints on the average number of RA and HVC_I_ bursts per song motif: *p*_R_ *L*_R_ ≅ 12 and *p*_I_ *L*_I_ ≅ 35.

**Figure 4:**
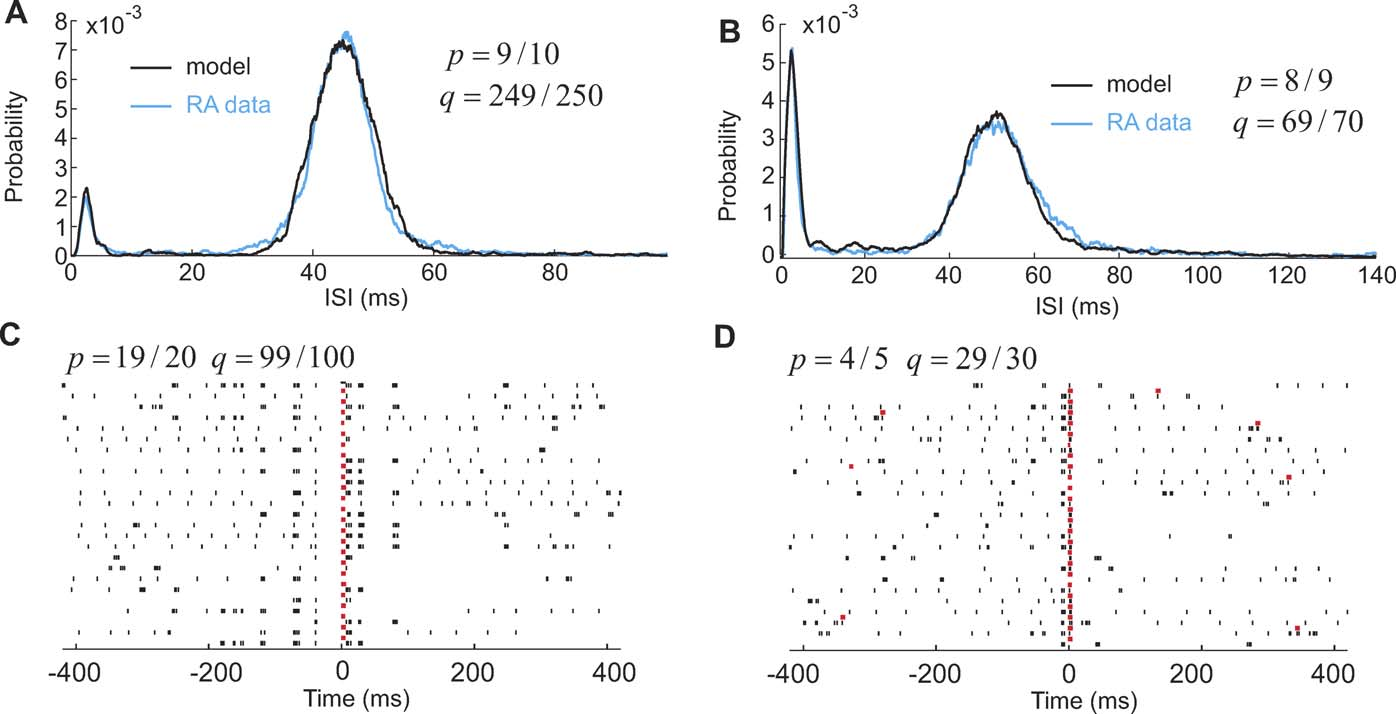
Modeling Sleep-Related Activity (*p,q <* 1) (A) An RA neuron producing few burst ISIs. A good fit is produced when the survival time of the ground state is long, compared to that of song states (light sleep, *q* much closer to 1 than *p*). *D*_R_ = 80 ms, and *V*_R_ = 0.7. (B) A different RA neuron producing many burst ISIs. A good fit was produced by a relatively long survival time of sleep states (deep sleep). *D*_R_ = 120 ms, and *V*_R_ = 0.67. (C,D) Spike raster plots of HVC_RA_ and RA neurons. All HVC_RA_ bursts (red rasters) are aligned at the center of the plots. Corresponding RA spikes (black rasters) are shown below each HVC_RA_ burst. When *p* is large (strongly coherent sleep) (C), stereotyped RA bursting is observed over larger intervals than when *p* is small (D).

Sleep-related ISI pdfs of RA neurons could be well-fit given a suitable tonic-firing model and suitable persistence probabilities *p* and *q* (Figure 4A and 4B). The peak at small ISIs resulted from spikes produced in song states, and the peak at large ISIs from spikes produced in the ground state. Raster plots of simulated RA-neuron activity aligned to HVC_RA_ bursts looked very realistic (compare Figure 4C and 4D to Figure 1Bi). Autocovariance functions of sleep-related RA spike trains could also be well-fit (see Figure S2).

The parameters *p* and *q* characterized what we shall refer to as the depth and the coherence of the sleep. By denoting the average number of time steps spent in song states by 〈*n_s_*〉 = *p* / (1 − *p*) and similarly 〈*n_a_*〉 = *p* / (1 − *q*) for the ground state (these numbers are known as the survival times in the language of point processes), we defined the sleep depth *d* by their ratio 〈*n_s_*〉 / 〈*n_a_*〉 (experimentally, *d* could be estimated from burst-rate measurements as *d* = *b* / (*b_0_* − *b*), where *b_0_* and *b* are measured burst rates during song and during sleep, respectively). Small ISIs prevailed during deep sleep (Figure 4B, 〈*n_s_*〉/ 〈*n_a_*〉 = 12%) and large ISIs during light sleep (Figure 4A, 〈*n_s_*〉/ 〈*n_a_*〉 = 3.6%). The coherence of sleep was defined by the product 〈*n_s_*〉〈*n_a_*〉 = 12%. Model ISI pdfs showed almost no dependence on sleep coherence. For example, by doubling both *p* and *q*, sleep-related ISI pdfs in Figure 4A and 4B remained essentially unchanged. However, the sleep coherence had a strong influence on raster plots: the larger the sleep coherence, the longer was the time interval relative to HVC_RA_ bursts over which stereotyped RA bursting could be observed (Figure 4C and 4D; note that sleep depths were very similar in Figure 4C and 4D: 19% versus 14%).

**Figure 5:**
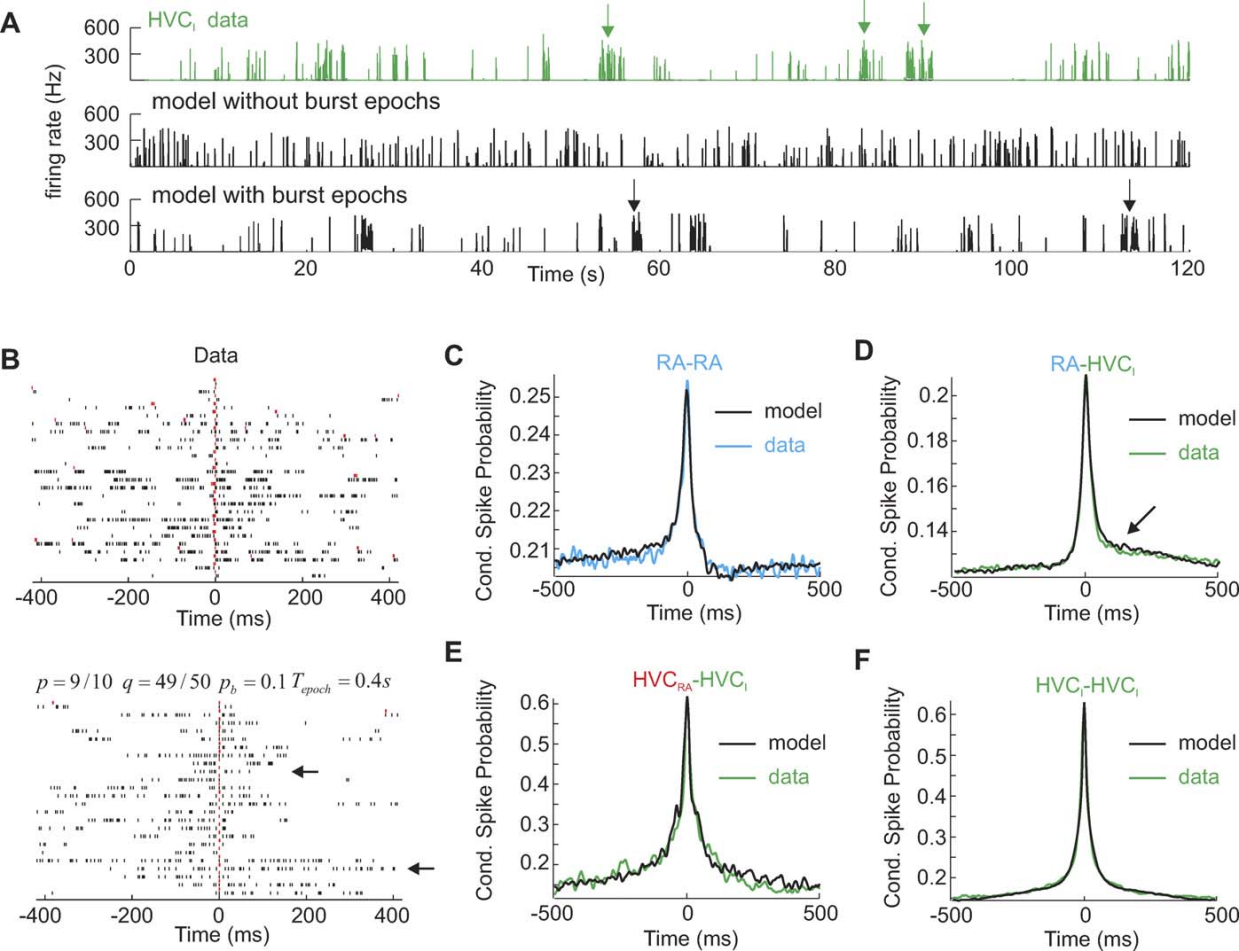
Burst Epochs and Pairwise Correlations (A) Instantaneous firing rates of a recorded HVC_I_ neuron (top), a simulated HVC_I_ neuron without burst epochs (middle), and a simulated HVC_I_ neuron with burst epochs (bottom). Burst epochs are indicated by arrows. (B) A sample raster plot of a simultaneously recorded HVC_RA_–HVC_I_ pair (top) and a comparable plot from model simulations (bottom). The inclusion of burst epochs gives rise to rows with very sparse HVC_I_ bursting (top arrow) and rows with dense HVC_I_ bursting (bottom arrow), as is seen in real data. (C–F) Average CSP functions in different neuron types. The functions are plotted in reference to a spike in the first pair, i.e., with respect to RA spikes in (D) and with respect to HVC_RA_ spikes in (E). (C) RA–RA neuron pairs (from *n* = 29 recorded pairs). *p* = 6/7, and *q* = 39/40. (D) RA–HVC_I_ pairs (*n* = 50 pairs). The arrow indicates an asymmetry that is reproduced by the model. *p* = 9/11, and *q* = 49/50. (E) HVC_RA_–HVC_I_ (*n* = 26). *p* = 7/8, and *q* = 59/60. (F) HVC_I_–HVC_I_ pairs (*n* = 19). HVC_I_ neurons randomly link to 56 of the 100 HVC_RA_ groups. *p* = 7/8, and *q* = 32/33. In (C–F) *L*_I_ = 50, *p*_I_ = 0.63, *D*_R_ = 240 ms, *p*_R_ = 0.92, and *L*_R_ = 13.

RA and HVC_I_ neurons frequently display 1–2 s epochs of increased burst density during sleep ([12]; Figure 5A, top). From a recent experimental study, we know that these burst epochs are shaped by input from the thalamic nucleus uveaformis (Uva): decreased tonic firing in HVC-projecting Uva neurons leads to increased bursting in HVC and RA neurons, whereas increased tonic firing in HVC-projecting Uva neurons suppresses HVC and RA burst rates (unpublished data). Here, we modeled this Uva-mediated control of burst epochs by random fluctuations of the parameter *p* (we transiently set *p* = 1 to model a burst epoch; see Methods) (Figure 5A, middle and bottom). By modifying *p* rather than any other parameter, we satisfied the experimental finding that burst shapes (burst-related ISI distributions) are unchanged during burst epochs. By virtue of burst epochs, raster plots of simulated HVC_RA_–HVC_I_ pairs were very realistic and displayed characteristic horizontal bands of long, uninterrupted bursting, coexisting with brief bands of very few bursts (Figure 5B). Without fluctuations in *p*, HVC_I_ burst patterns would mostly be either narrow or wide, but not both.

One of the touchstones of our model is whether it can reproduce pairwise correlations in sleep-related spike trains on large time scales (two orders of magnitude beyond the burst scale). We modeled CSP functions by averaging over 50 simulated cell pairs with randomly drawn link sets. It was a simple matter to produce excellent fits of CSP functions in RA–RA and RA–HVC_I_ pairs (Figure 5C and 5D). The effect of *p* was to set the width of CSP functions, whereas *q* and average RA and HVC_I_ firing rates set the baseline and peak values. However, CSP function in HVC_RA_–HVC_I_ pairs and HVC_I_–HVC_I_ pairs turned out to be more problematic because it was impossible to reproduce the high CSP peaks near zero time lag. For HVC_RA_–HVC_I_ pairs, there was a simple explanation for this shortcoming: when we simulated only as many model pairs as were available in the experimental dataset (26 instead of 50), then the high CSP peak could be occasionally reproduced due to random link sampling (Figure 5E). Thus, from a bootstrapping point of view, the small difference between model and real CSP functions in HVC_RA_–HVC_I_ pairs was not statistically significant. In contrast, the peak CSP in HVC_I_–HVC_I_ pairs was significantly higher than its model counterpart: even when sleep activity was restricted to song states only (*p* = 1), the high peak CSP in HVC_I_–HVC_I_ pairs could not be reproduced. A good fit was only possible with substantially higher HVC_I_ burst probability, *p*_I_ = 0.95. Thus, we were faced with the paradoxical conclusion that HVC_I_ neurons burst more reliably during sleep than during singing (this conclusion is paradoxical, because with our estimate of HVC_RA_ burst probability *p*_P_ = 1 during singing and *p*_P_ = 0.8 during sleep, the presumed HVC_RA_ drive is smaller during sleep, and so *p*_I_ should be smaller as well). We could imagine two reasons why the CSP peaks of HVC_I_ pairs might be so high during sleep. First, during sleep, HVC_I_ neurons could be selectively driven by X-projecting HVC (HVC_X_) neurons or by neurons in the nucleus interface of the nidopallium (NIf) that project to HVC (NIf_HVC_ neurons), in addition to their weaker drive from HVC_RA_ neurons. This explanation by itself seems somewhat implausible, because it would require that HVC_RA_ neurons not be driven (or only very weakly driven) by HVC_X_ or NIf_HVC_ neurons, which appears not to be the case [21–23]). Therefore, we favored a second explanation, which is that our assumption of random and uniform links in HVC_I_ neurons must be wrong. In other words, there must be a special subset of HVC_RA_ groups to which HVC_I_ neurons are linked with higher probability. In fact, such an explanation agrees with song-related data, according to which HVC_I_ population activity is weakly correlated with sound amplitude and therefore not uniformly distributed over the time course of a song motif [11]. Indeed, when we relaxed the assumption that HVC_I_ neurons can be linked to any one of the 100 HVC_RA_ groups, but to only 56 randomly selected groups, we obtained a good fit to the CSP peak with standard HVC_I_ parameters *p*_I_ = 0.63 (*L*_I_ = 50) (Figure 5F).

Note that a requirement for the excellent CSP fits was the inclusion of burst epochs. Without burst epochs, the long tails of CSP functions could not be well fit (see Figure S3). Note also that the asymmetry in the average RA–HVC_I_ CSP function in Figure 5D was largely due to RA inhibition that decreases tonic firing after bursts and due to differences between RA and HVC_I_ tonic firing rates.

One of our model assumptions is that any HVC_RA_ group can be activated from within the ground state. We were unable to stringently test this assumption: All of our results remained unchanged when singing-like activity could be initialized in only a random subset of ten or more song states. However, when this number was much smaller (two to four states), unrealistic peaks in correlation functions appeared, thereby setting a lower bound for the number of possible initial HVC_RA_ groups.

### Tests of HVC Ultrasparseness and Sequential Dynamics during Sleep

We tested the validity of our assumptions of ultrasparseness and sequential dynamics of HVC_RA_ activity. Given that during sleep HVC_RA_ bursts are time-locked to burst patterns in RA neurons (Figure 1Bi), we decided to use this locking to test whether individual HVC_RA_ neurons are linked to a single or, potentially, to several HVC_RA_ groups, and whether during sleep, HVC_RA_ groups are activated sequentially or in more random order.

**Figure 6:**
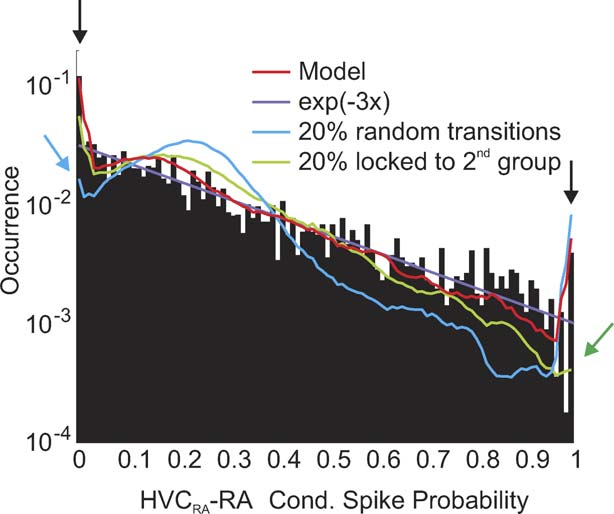
HVC States Evolve Sequentially and Are Formed by Distinct HVC_RA_ Groups Distribution of CSPs in (*n* = 46) HVC_RA_–RA pairs in the interval −60 to 60 ms of HVC_RA_ spikes (black histogram). With the exception of two peaks at CSPs zero and one (black arrows), the distribution is well-approximated by an exponential curve (purple line). Shown are the average CSP functions of 50 simulated HVC_RA_–RA pairs for three different model assumptions: (1) HVC_RA_ neurons fire with probability *p*_P_ = 0.8 in a single HVC_RA_ group (red curve); (2) HVC_RA_ neurons fire in two (randomly selected) HVC_RA_ groups with probabilities 0.64 and 0.16 (green curve); and (3) activation of HVC_RA_ groups is sequential in 80% of song-like transitions and in 20% it is random (blue curve). The green and blue arrows indicate inadequacies of model assumptions 2 and 3. *p* = 6/7, *q* = 39/40, *L*_R_ = 12, *p*_R_ = 1, *D*_R_ = 240 ms, and *p*_b_ = 0.

We determined the experimental CSP distribution of all HVC_RA_–RA pairs in the time interval [−60, 60] ms of HVC_RA_ spikes (Figure 6). With the exception of extreme (very small and very large) CSPs, the distribution was well-approximated by an exponential curve. The excessive occurrence of extreme CSPs did not happen by chance: the number of CSPs in the bin [0.99, 1] was significantly larger than the number of CSPs in equally sized adjacent bins (*p* < 0.01, binomial test). The same held true for the number of CSPs in the bin [0, 0.01], which was significantly larger than in adjacent bins. This CSP behavior illustrates that on the population level, RA activity tends to be highly locked to HVC_RA_ bursts within at least ±60 ms.

We compared the experimental CSP distributions with model distributions for 50 simulated HVC_RA_–RA pairs under various model assumptions. For the model in Figure 2, very small and very large CSPs appeared frequently (red curve in Figure 6), in good agreement with the data. Almost no parameter tuning was necessary to achieve a good fit. The heights of extreme CSP peaks were positively correlated with *q*. When *q* was small, the likelihood of repeated switching between ground and song states within 60 ms was large, thereby decorrelating spike trains and forcing extreme CSP values to appear less frequently. CSPs in the intermediate range 0.5–0.95 were positively correlated with *p*, because with longer RA burst sequences, intermediate CSP values occurred more often. For a peak at unit CSP to appear, the RA burst probability *p*_R_ had to be close to one: by decreasing *p*_R_ from one to 0.8, the peak at unit CSP completely disappeared. Thus, to agree with the data, RA neurons must have a very high burst probability, which is suggestive of a strong drive from HVC.

We then assumed that HVC_RA_ neurons do not burst by linkage with a single HVC_RA_ group, but that 80% of their bursts are locked to a first HVC_RA_ group, and 20% of bursts are locked to a second group (in the simulations, the two groups were randomly chosen for each simulated HVC_RA_ neuron). We expected these double linkages to create a washout effect in which clear RA burst pattern would no longer be seen. Indeed, by remapping just 20% of HVC_RA_ bursts in this manner, very high and very low CSPs appeared less frequently (green curve in Figure 6), in disagreement with the data. This phenomenon was very robust because increasing *p* up to 99/100 and *q* up to 999/1000 was insufficient to reproduce the high peak at unit CSP. Thus, ultrasparseness of HVC_RA_ linkage is necessary to explain the abundance of extreme CSPs.

We also estimated the degree to which HVC_RA_ groups are activated in sequence as opposed to random (nonsequential) activation. In principle, our sleep model in Figure 2B allows for almost arbitrary state transitions by means of a brief intermission via the ground state. However, reasonable values for *p* and *q* imply that nonsequential HVC_RA_-group activation is rare and that such events have little impact on the observed CSP distribution. To test our model assumption that fixed and sequential state transitions underlie sleep-related activity patterns, we performed simulations in which 20% of transitions between song states were nonsequential but completely random. We found that introducing such randomness into the model resulted in an altered CSP distribution in which zero CSPs appeared very infrequently, in stark disagreement with the data (blue curve in Figure 6). The reason for this lack was that with increasing time lag to HVC_RA_ spikes, stray RA bursts started to appear due to random transitions, leading to non-zero CSPs. This phenomenon was very robust as it was not possible to remedy the scarcity of zero CSPs by changing *p* and *q.* Note that the peak at unit CSP was unchanged by the introduction of random transitions, presumably because unit CSPs arose only at very small time lags away from HVC_RA_ spikes and thus were not significantly affected by the random transitions. In conclusion, the inclusion of few nonsequential transitions leads to severe decorrelation of RA spikes a few tens of milliseconds away from HVC_RA_ spikes and to shortage of very small CSPs; therefore, nonsequential transitions of HVC activity must be very rare during sleep. Note that by the same argument, we could also exclude the possibility that two or more HVC-activation sequences can coexist at the same time. If this were the case, then extreme CSPs would be rare, even more so than by relaxing sequential order or ultrasparseness.

### Tests of RA Intrinsic Dynamics and Inhibition

**Figure 7:**
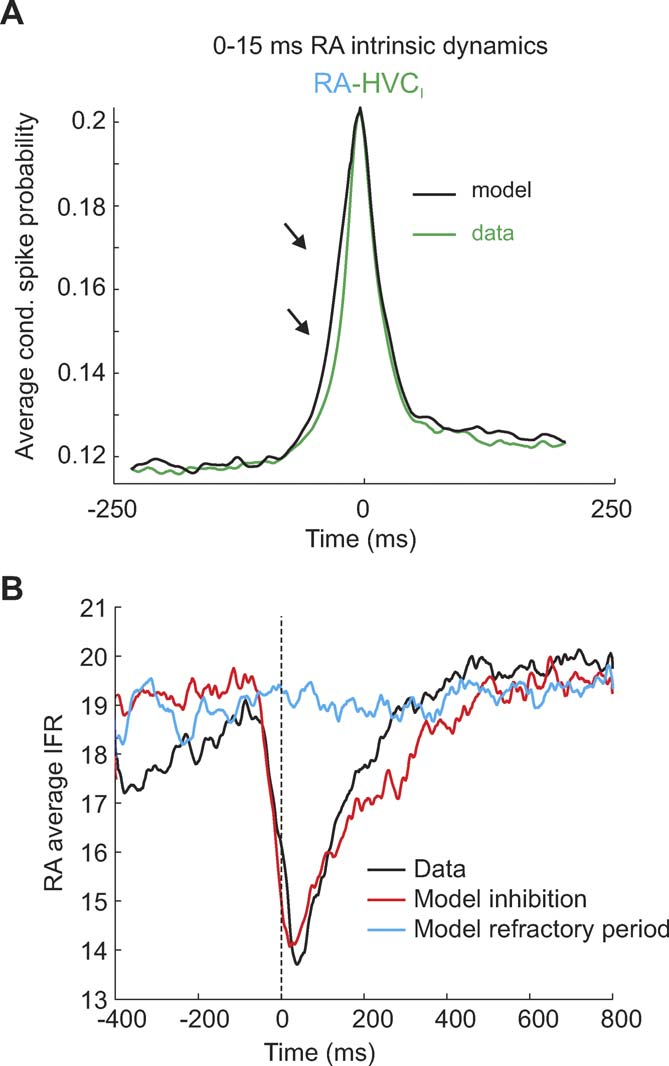
RA-Intrinsic Dynamics and Inhibition (A) When RA burst sequences extend beyond HVC_I_ sequences by a random time uniformly distributed in the interval 0–15 ms, then the left flank of the average RA–HVC_I_ CSP function gets uncharacteristically wide (arrows). (B) Transitive suppression of tonic firing in RA neurons is explained by RA inhibition. Shown are average RA IFR curves in 1.2 s time windows in which one RA neuron does not fire a burst, and time-aligned to burst onset in a simultaneously recorded RA neuron. Conjunctively with the bursts, there is a transient reduction in firing rate of the nonbursting neuron (black curve, *n* = 50 RA neuron pairs). The model in which RA inhibition suppresses spontaneous firing (red curve) is able to reproduce this transient reduction, but the model in which RA neurons display a soft refractory period after bursts (blue curve) is not. *p* = 6/7, *q* = 39/40, *L*_R_ = 12, *p*_R_ = 1, *D*_R_ = 240 ms, and *p*_b_ = 0.

In our model, RA neurons are simply driven by HVC_RA_ bursts. To test for the possibility that RA burst sequences can be self-sustaining due to recurrent RA circuitry and in the absence of HVC drive, we performed model simulations in which after each transition into the ground state, RA burst sequences continued to propagate for a random duration uniformly distributed in the time interval 0–15 ms. By doing this, RA neurons produced less than 4% additional burst spikes compared to before. Despite this small addition of spikes, average CSP functions of RA–HVC_I_ pairs became unrealistically heavy at negative time lags, Figure 7A. This behavior was very robust, though it obviously depended on the estimated HVC_RA_ spike propagation time *t*_R_ = 4 ms; see Methods and [24]. To assess the relevance of RA intrinsic dynamics in a manner independent of spike-propagation estimates, we removed single spikes in RA neurons (these are spikes forming ISI pairs of more than 10 ms each). Thus-formed RA–HVC_I_ CSP functions (with single RA spikes removed) displayed a high peak that in fact could not be reproduced with any set of model parameters *p* and *q* unless RA links were correlated with HVC_I_ links (good agreement could be achieved when RA neurons were linked to 13 among the 56 HVC_RA_ groups to which HVC_I_ neurons were linked). Thus, rather than finding evidence for RA intrinsic dynamics, we found the contrary evidence that in order to explain the non-lagging and strong RA–HVC_I_ correlations, RA neurons must be preferentially linked to and driven by the same HVC_RA_ groups as are HVC_I_ neurons.

We were also able to test a more subtle prediction of our model, such as the impact of RA-intrinsic inhibition. The key experimental observation is that right after sleep bursts, RA neurons do not immediately reenter the tonic firing mode, but that tonic firing recovers after an estimated recovery time of *D*_R_ = 240 ms (Figure S1E). We modeled this transient suppression of tonic firing by RA inhibition. This inhibition had average duration *D*_R_ and was randomly elicited with independent probability *P*_Inh_ per activated HVC_RA_ group (see Methods). A good fit was achieved using *P*_Inh_ = 0.1. Due to the nonspecificity of this inhibition, tonic RA firing was suppressed also when the recorded RA neuron did not burst, but some other RA neuron did. The situation was different when we modeled the reduced tonic firing by a soft refractory period with average duration *D*_R_ = 240 ms, in which case tonic RA firing was suppressed only after bursts. To distinguish between these two models, we inspected paired RA–neuron recordings for periods when one neuron burst, but the other did not. We then plotted the average instantaneous firing rate (IFR) of the nonbursting neurons, time-aligned to burst onsets. We found that in synchrony with the bursts, there was a brief dip in the IFR. The inhibition model was able to reproduce this phenomenon, but the adaptation model was not, Figure 7B. These findings demonstrate that tonic RA firing during sleep is suppressed by intrinsic inhibition and not by firing adaptation alone.

## Discussion

We have translated a popular diagram of songbird premotor dynamics into a simple state-space model of neuron populations. To produce good fits of spike correlations measured during sleep, we had to make use of a nonnegligible range of parameter values. We justified this requirement by intrinsic variability of the data that on the one hand is due to nonstationarities of sleep modeled by *p* and *q*, and on the other hand is due to individual differences in tonic firing rates. The parameters *p* and *q* interpolate between firing characteristics associated with two different behavioral states, i.e., waking and singing. We can at this point only speculate about their biophysical interpretations.

The persistence probability *p* of song states could be a neuromodulatory mechanism that affects vesicle release probability in HVC_RA_ neurons, or their excitability. Such a scenario seems plausible if sequential activation of HVC_RA_ groups derives from excitatory synaptic connections between HVC_RA_ neurons. Current evidence indicates that HVC and RA burst epochs are shaped by a thalamic nucleus. Accordingly, the persistence probability *p* must depend on such extrinsic influences as well. We are more uncertain about the persistence probability *q* of the ground state. Songs of birds are initiated somewhere in the brain with the result of activating a particular HVC_RA_ group. During sleep, initializing signals appear to originate in the NIf that projects to HVC [23]. The parameter *q* could thus represent vesicle release probability in synapses of HVC-projecting NIf neurons or of synapses (or excitability) within NIf.

An inherent assumption in our model is conditional independence of spike trains given a sequence of population states. This is a strong assumption, as it ignores the fact that cells spike more reliably when their afferents spike more reliably as well. As a consequence, we found that the model tended to underestimate some measured correlations (Figure 5F), yet the differences could be explained by assuming nonhomogeneity of link distributions. Possibly, by doing so, we have overestimated the tendency by which neurons link to preferred HVC_RA_ groups; part of the high CSPs could be attributable to genuine pairwise interactions. To be able to estimate these interactions in future work, it will be necessary to simultaneously record from larger neuron populations. Our prediction would be that higher-order spike correlations must obey the regularities imposed by population-conditional spike-generation mechanisms. If this prediction turns out to be wrong and spike triplets appear more often than predicted, then we might have to revise our model by incorporating mutual dependencies of burst probabilities, which in essence corresponds to introducing higher-order spike correlations.

We were unable to characterize the HVC_RA_ groups to which HVC_I_ and RA neurons are linked with higher probability, but we speculate that preference applies to HVC_RA_ groups that represent syllable onsets, in agreement with weak predictive correlations between song patterns and activity in HVC_I_ and RA neurons [10,11]. These distinguished HVC_RA_ groups could also be leaders that are preferentially activated in transition from the ground state. Such a scenario seems plausible given that syllable onsets are flexible song elements optimally aligned with global song tempo [25]. Insights into these questions could emerge from applications of our modeling approach to a set of HVC and NIf recordings [23]: because NIf projection neurons tend to burst in time intervals of 100 ms and more, their correlations with HVC neurons might provide evidence of regular spacing between leading HVC_RA_ groups.

One of the benefits of our modeling approach compared to other approaches is increased simulation efficiency, because the time it takes to generate a model spike train is orders of magnitude shorter than for detailed biophysical models such as conductance-based integrate-and-fire neurons. Thanks to this efficiency, we were able to compare simulated data with real data to great detail, a task that usually becomes exhaustive in simulations of membrane biophysics. We have not hand-picked neurons for model comparison, but tested model predictions on data from all recorded cells and in all relevant behavioral states. Despite the many simplifications of our model, we believe it can be converted into the language of membrane voltages and synaptic potentials. For example, we have implicitly assumed that neurons are intrinsic bursters. It is known that intrinsic bursting can stabilize the propagation of synchronized activity in conductance-based model neurons [14]. One of the main difficulties would then be to find the appropriate conductance values that implement our estimates of burst probabilities and burst durations. In contrast, comparatively little effort would have to be made to compose synaptic weight matrices, as these are specified by our estimates of link statistics.

It might also be interesting to apply our approach to other neural systems. For example, in the insect olfactory system, odor processing is associated with stereotyped neural sequences in the antennal lobe [26]. Although the diversity of these sequences is thought to have the function of maximizing odor discriminability in downstream areas, it is currently not clear whether odor-evoked sequences are assembled from discrete states and constrained by a small number of state transitions, or whether an almost infinite number of possibilities applies [27]. Our approach would be ideally suited to explore such hypotheses.

Our findings suggest that all sleep-related bursts are in fact replay of song-evoked bursts, as each model sleep burst is clearly associated with one of 100 song-related activity states. Such similarity seems not surprising given that song- and sleep-related activity is generated by the same synaptic circuits. However, what could be the function of such randomized replay? We do not know the answer, but generative probabilistic models as ours have the advantage that they are closely related to some machine learning algorithms [28]. With the growing notion that activity replay during sleep may be involved in memory consolidation and learning processes [29,30], our model provides a sound basis for the quantitative testing of such ideas.

## Methods

### Markov population model of HVC activity.

We model the activity state of HVC at time *t* as a random variable *S_t_* that can be in any one of 101 states, where state 0 is termed the ground state and states 1–100 are termed song states (Figure 2). When at time *t* the random variable is in the *i* ^th^ song state (*S_t_* = *i* > 0), we say that the *i* ^th^ group of HVC_RA_ neurons (or *i* ^th^ HVC_RA_ group) is activated. Accordingly, at time *t* + δ*_t_*, the (*i* + 1)^th^ group is activated with probability *p* (a free model parameter): 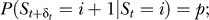 alternatively, with probability 1 − *p*, HVC activity transits into the ground state: 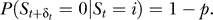 The space of song states has a ring structure such that when the 100^th^ state is reached, HVC activity transits into state 1 with probability *p.* When at time *t*, HVC activity is in the ground state, it stays there at time *t* + δ*_t_* with probability *q* (another free model parameter): 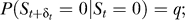 alternatively, with probability 1 − *q*, HVC activity transits into any one of the song states, 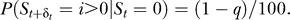


The time steps δ*_t_* in which HVC dynamics evolve is a random variable that depends on the HVC_RA_ group that is active at that time: 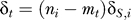 . Here, *n_i_* sets the fixed maximum time-step duration of the *i* ^th^ group (a Gaussian random number with a mean of 9 ms and standard deviation of 1.8 ms), *m_i_* introduces temporal fluctuations (a Gaussian random variable with a mean 4 ms and standard deviation 0.4 ms), and 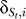 is the Kronecker delta (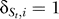 if *S_t_* = *i*, and 0 otherwise). The reason for this doubly random choice of time steps is to avoid any periodicity which would lead to uncharacteristic ultranarrow peaks in correlation functions. For the ground state, time steps are not randomized, but simply set to the average duration of song states, i.e., 5 ms. The large-time behavior of the model output was independent of detailed time-step assumptions.

### Model spike trains.

Given a sequence {*S_t_*}_0<*t*<*T*_ of HVC activity states, we generate spike trains in a small set of HVC_RA_, HVC_I_, and RA neurons in the following manner. First, we randomly link each of the neurons to a distinct subset of HVC_RA_ groups, where the subset size (the link count) ranges from *L*_I_ = 35 to 50 for HVC_I_ neurons, from *L*_R_ = 12 to 13 for RA neurons, and is set to 1 for HVC_RA_ neurons. In the time interval [*t*, *t* + δ*_t_*], neuron X is (1) in the *burst mode* with probability *p*_X_, if *S_i_* = *i* > 0 and if neuron X is linked to the *i* ^th^ HVC_RA_ group, or (2) in the *tonic* (*firing*) *mode* otherwise (X = P for HVC_RA_ neurons, X = R for RA neurons, and X = I for HVC_I_ neurons).

For HVC_RA_ neurons, we chose *p*_P_ = 1 during singing and *p*_P_ = 0.8 during sleep [1], though none of the results depended on the actual value of *p*_P_ (due to our conditional assessment of spike correlations).

Spikes associated with the two firing modes are generated by time rescaling of a Poisson process [20] using *conditional intensity functions* (CIFs). The CIF *h*(τ) (also known as the stochastic intensity function) is the instantaneous spiking probability as a function of the time lag τ since the last spike. Mathematically, the CIF is defined by the conditional probability *h*(*τ*) = *P*(one spike in [*t* + *τ*, *t* + *τ* + Δ*t*]| last spike at *t*), where Δ*t* = 0.1 ms is the smallest time unit in our simulations. CIFs can be derived from ISI pdfs *p*(τ) according to [20]:

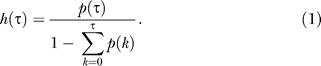



The CIFs associated with burst modes are denoted by *h^b^*(τ) (*b*, as in burst) and are identical for all neurons of a given type; they are derived from averages of measured ISI pdfs (Figure 2C). During sleep, firing rates of burst spikes are typically lower than during singing (see Figure S1 for a comparison), suggesting a weakened synaptic drive during sleep. To account for this fact, when modeling sleep behavior (*p* < 1 and *q* < 1), we sample burst CIFs *h^b^*(τ) at a reduced speed defined by *h^b^*(*V*τ), where *V*_P_ = 0.63 for HVC_RA_ neurons, *V*_R_ = 0.65 for RA neurons, and *V*_I_ = 0.9 for HVC_I_ neurons. The CIFs associated with tonic firing modes in HVC_I_ and RA neurons are denoted by *h^a^*(τ) (*a*, as in awake) and are modeled by gamma functions (Figure 2D).

To model spike propagation times from HVC to RA [1,24], we add a fixed delay of 4 ms to all RA spikes. By construction, spike trains restricted to time intervals [*t*, *t* + δ*_t_*] have renewal statistics, but because of frequent state switching of the HVC population, renewal statistics does not apply to large time intervals. All our simulations are performed with a unit time step of Δ*t* = 0.1 ms. For each simulated neuron, we generate spike trains between 2 min and 30 min duration.

The following additional assumptions about switching behavior produce good results: when a neuron switches from the tonic mode into the burst mode, we automatically set the first spike of the burst. However, if a neuron remains for two or more consecutive time steps in the burst mode, then we continue to sample the CIF without setting a spike at subsequent time steps (we set a spike only after a state switch).

To model reduced tonic firing in RA neurons after spike bursts (Figure S1E), we incorporate an RA inhibitory mechanism into the model. We assume that in each song state, there is an (independent) probability *P*_Inh_ = 0.1 that a neuron experiences inhibitory input from RA interneurons [31]. Such inhibitory input lasts for a duration *D*, where *D* is randomly drawn from an exponential distribution with mean *D*_R_. As long as an RA neuron receives inhibitory input, it does not produce tonic spikes (in contrast, RA neurons are allowed to fire burst spikes while subjected to inhibitory input). To test the validity of this inhibition model, we compare it to a different model in which tonic firing is reduced after bursts by means of burst-triggered spike-rate adaptation. That is, when an RA neuron switches into the tonic firing mode, no spike is fired until a random time delay *D* passes since the onset of the last burst, where *D* is again randomly drawn from an exponential distribution with mean *D*_R_. Both models are able to explain burst-triggered firing adaptation in single RA neurons (Figure S1E); however, only the inhibition model is able to correctly reproduce transitive firing suppression in RA pairs (Figure 7).

Bursting in RA and HVC neurons is under tight control of input from Uva (unpublished observation). We implement Uva-mediated burst epochs as a Poisson point process: in regular time intervals of *T*_epoch_ = 400 ms and with probability *p_b_* = 0.04, we increase the persistence of song states to *p* = 1 for a duration of *T*_epoch_. No fine-tuning of burst epoch parameters *T*_epoch_ and *p_b_* was necessary to produce good fits in Figure 5C–5F.

Curves in Figures 2 to 5 were fit by manual parameter selection using a graphical user interface written in MATLAB (The Mathworks) and C++. The parameter values that were explored to produce fits in Figures 4 and 5 were *p*, *q*, and the average tonic firing rates in RA and HVC_I_ neurons (fixed for each neuron type). No objective fitting criterion or systematic parameter sampling was used; satisfactory results could be obtained by trial and error.

### Spike-train analysis.

All spike-train analysis is performed using Matlab scripts mixed with fast C++ routines. Methods are described in detail in [12] and [24].

The IFR *R*(*t*) is defined as the inverse of the ISI enclosing time *t*.

The ISI pdf *p*(*τ*) (*τ* is the ISI) is defined as the histogram of ISIs normalized to sum to one.

We estimate CSP functions *P*_B|A_(*t*) for simultaneously recorded or simulated neuron pairs A and B in terms of the fraction of spikes in neuron A that are associated with at least one spike in neuron B in the relative time window [*t* − ½*s*, *t* + ½*s*]:

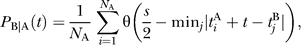
where *N_A_* is the total number of spikes in neuron A, 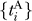 are the spike times of neuron A, 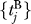 are the spike times of neuron B, 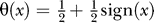 is the Heavyside function, and *s* = 5 ms is the halfwidth of the spike clipping window. For more information on CSP functions, consult [24].

## Supporting Information

Figure S1 Comparison of Song-Related and Sleep-Related Average ISI pdfs (A–D) Shown are ISI pdfs (normalized to the first 10 ms) measured during singing and during sleep. In all neuron types, sleep-related bursts have lower firing rates, indicated by the rightward shift of ISI peaks. Matching of singing-related and sleep-related ISI pdfs can be achieved by different stretch factors *V* (see Methods). *V* = 0.65 for RA neurons in (A), *V* = 0.9 for HVC_I_ neurons in (B), *V* = 0.63 for HVC_RA_ neurons in (C), and *V* = 0.77 for X-projecting HVC neurons (HVC_X_ neurons) in (D). ISI pdfs were produced based on data in [1,10–12].
(E) RA spike histogram for a range of time lags since the last sleep burst, computed for all RA bursts that were followed by a burst-free period of at least 2 s (the histogram is composed of RA single spikes only). The red curve depicts the fit 1.9 − 1.3exp(*t/D*_R_), where *t* is the time lag since the last burst, and *D*_R_ = 240 ms is our estimation of the RA inhibition time constant.
(94 KB PDF)

Figure S2Autocovariance Functions of RA Spike Trains during SleepThe autocovariance function *C*(*t*) of a spike train *ρ*(*t*) (modeled as a sum of delta functions) is a measure of spike density fluctuation and is defined as

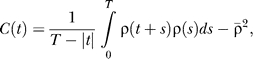
where 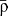 is the average firing rate and *T* is the total duration of the spike train. The characteristic oscillatory behavior of autocovariance functions in RA neurons is well-reproduced by the model.
(A) A short survival time of the ground state leads to fast decay of autocovariance oscillations. *D*_R_ = 240 ms and *V*_R_ = 0.7.(B) A long survival time of the ground state leads to slow decay of oscillations. *D*_R_ = 120 ms and *V*_R_ = 0.67.In (A) and (B), *L*_R_ = 13 and *p*_R_ = 0.92.(85 KB PDF)

Figure S3 Average CSP Functions Fitted without Burst Epochs (A–D) Unlike in Figure 5, no burst epochs (fluctuations in *p*) were included in the model. Model curves (black) represents the best fits achievable by trial and error. The arrows indicate regions where the quality of fit could not be improved. Same legend as in Figure 5.
(49 KB PDF)

## Abbreviations

CIF: conditional intensity function
CSP: conditional spike probability
HVC: high vocal center
IFR: instantaneous firing rate
ISI: interspike interval
NIf: nucleus interface of the nidopallium
pdf: probability density function
RA: robust nucleus of the arcopallium
Uva: thalamic nucleus uveaformis

## References

[pcbi-0030249-b001] Hahnloser R, Kozhevnikov A, Fee MS (2002). An ultrasparse code underlies the generation of neural sequences in a songbird. Nature.

[pcbi-0030249-b002] Luczak A, Bartho P, Marguet SL, Buzsaki G, Harris KD (2007). Sequential structure of neocortical spontaneous activity in vivo. Proc Natl Acad Sci U S A.

[pcbi-0030249-b003] Kenet T, Bibitchkov D, Tsodyks M, Grinvald A, Arieli A (2003). Spontaneously emerging cortical representations of visual attributes. Nature.

[pcbi-0030249-b004] Dave AS, Margoliash D (2000). Song replay during sleep and computational rules for sensorimotor vocal learning. Science.

[pcbi-0030249-b005] Louie K, Wilson MA (2001). Temporally structured replay of awake hippocampal ensemble activity during rapid eye movement sleep. Neuron.

[pcbi-0030249-b006] Tsodyks M, Kenet T, Grinvald A, Arieli A (1999). Linking spontaneous activity of single cortical neurons and the underlying functional architecture. Science.

[pcbi-0030249-b007] Dan Y, Alonso JM, Usrey WM, Reid RC (1998). Coding of visual information by precisely correlated spikes in the lateral geniculate nucleus. Nat Neurosci.

[pcbi-0030249-b008] Hatsopoulos NG, Paninski L, Donoghue JP (2003). Sequential movement representations based on correlated neuronal activity. Exp Brain Res.

[pcbi-0030249-b009] Schneidman E, Berry MJ, Segev R, Bialek W (2006). Weak pairwise correlations imply strongly correlated network states in a neural population. Nature.

[pcbi-0030249-b010] Leonardo A, Fee MS (2005). Ensemble coding of vocal control in birdsong. J Neuroscience.

[pcbi-0030249-b011] Kozhevnikov A, Fee MS (2006). Singing-related activity of identified HVC neurons in the zebra finch. J Neurophysiol.

[pcbi-0030249-b012] Hahnloser HR, Kozhevnikov AA, Fee MS (2006). Sleep-related neural activity in a premotor and a basal-ganglia pathway of the songbird. J Neurophysiol.

[pcbi-0030249-b013] Dave AS, Yu AC, Margoliash D (1998). Behavioral state modulation of auditory activity in a vocal motor system. Science.

[pcbi-0030249-b014] Jin DZ, Ramazanoglu FM, Seung HS (2007). Intrinsic bursting enhances the robustness of a neural network model of sequence generation by avian brain area HVC. J Comput Neurosci.

[pcbi-0030249-b015] Fee MS, Kozhevnikov AA, Hahnloser RH (2004). Neural mechanisms of vocal sequence generation in the songbird. Ann N Y Acad Sci.

[pcbi-0030249-b016] Deppisch J, Pawelzik K, Geisel T (1994). Uncovering the synchronization dynamics from correlated neuronal activity quantifies assembly formation. Biol Cybern.

[pcbi-0030249-b017] Danoczy M, Hahnloser HR (2005). Efficient estimation of hidden state dynamics from spike trains. Neural Inf Process Syst (NIPS) 18.

[pcbi-0030249-b018] Yu BM, Afshar A, Snathanam G, Ryu SI, Shenoy KV (2005). Extracting dynamical structure embedded in neural activity. Neural Inf Process Syst (NIPS) 18.

[pcbi-0030249-b019] Truccolo W, Eden UT, Fellows MR, Donoghue JP, Brown EN (2005). A point process framework for relating neural spiking activity to spiking history, neural ensemble, and extrinsic covariate effects. J Neurophysiol.

[pcbi-0030249-b020] Brown EN, Barbieri R, Ventura V, Kass RE, Frank LM (2002). The time-rescaling theorem and its application to neural spike train data analysis. Neural Comput.

[pcbi-0030249-b021] Rosen MJ, Mooney R (2006). Synaptic interactions underlying song-selectivity in the avian nucleus HVC revealed by dual intracellular recordings. J Neurophysiol.

[pcbi-0030249-b022] Mooney R, Prather JF (2005). The HVC microcircuit: the synaptic basis for interactions between song motor and vocal plasticity pathways. J Neurosci.

[pcbi-0030249-b023] Hahnloser RH, Fee MS (2007). Sleep-related spike bursts in HVC are driven by the nucleus interface of the nidopallium. J Neurophysiol.

[pcbi-0030249-b024] Hahnloser RH (2007). Cross-intensity functions and the estimate of spike-time jitter. Biol Cybern.

[pcbi-0030249-b025] Glaze CM, Troyer TW (2006). Temporal structure in zebra finch song: implications for motor coding. J Neurosci.

[pcbi-0030249-b026] Mazor O, Laurent G (2005). Transient dynamics versus fixed points in odor representations by locust antennal lobe projection neurons. Neuron.

[pcbi-0030249-b027] Rabinovich M, Volkovskii A, Lecanda P, Huerta R, Abarbanel HD (2001). Dynamical encoding by networks of competing neuron groups: winnerless competition. Phys Rev Lett.

[pcbi-0030249-b028] Hinton GE, Dayan P, Frey BJ, Neal RM (1995). The “wake-sleep” algorithm for unsupervised neural networks. Science.

[pcbi-0030249-b029] Deregnaucourt S, Mitra PP, Feher O, Pytte C, Tchernichovski O (2005). How sleep affects the developmental learning of bird song. Nature.

[pcbi-0030249-b030] Walker MP, Stickgold R (2006). Sleep, memory, and plasticity. Annu Rev Psychol.

[pcbi-0030249-b031] Spiro JE, Dalva MB, Mooney R (1999). Long-range inhibition within the zebra finch song nucleus RA can coordinate the firing of multiple projection neurons. J Neurophysiol.

