# Supplementary figures and images for: Spike Correlations in a Songbird Agree with a Simple Markov Population Model
Source: PLoS Comput Biol. 2007 Dec 21;3(12):e249. doi: 10.1371/journal.pcbi.0030249 (PMC2230679; doi:10.1371/journal.pcbi.0030249)

**A**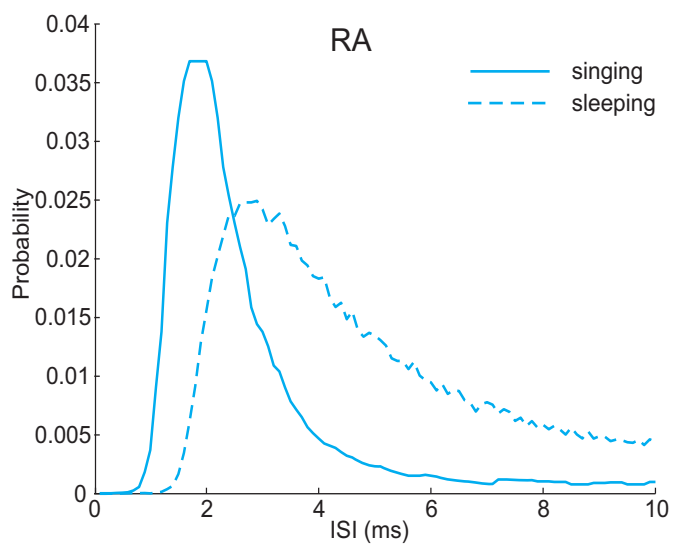**B**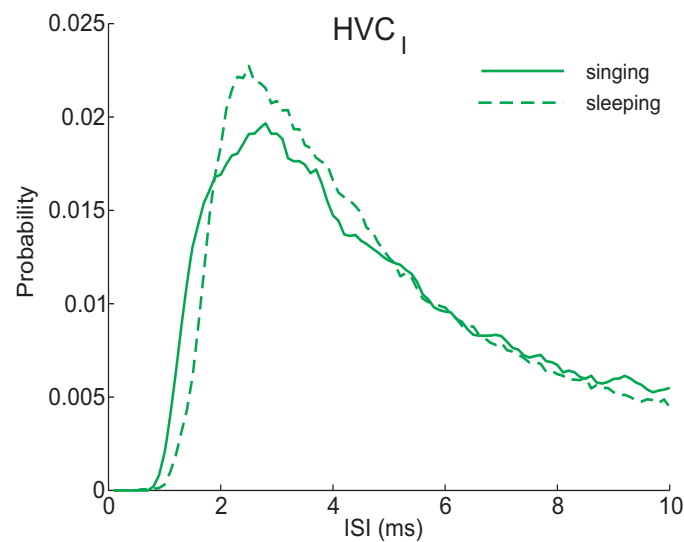**C**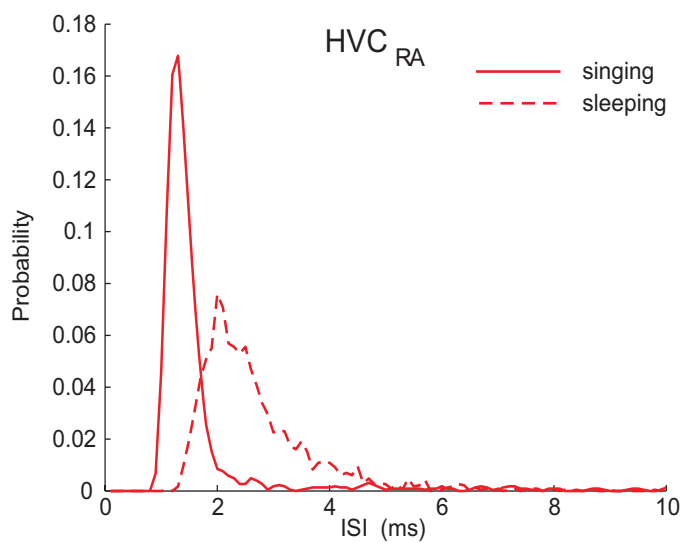**D**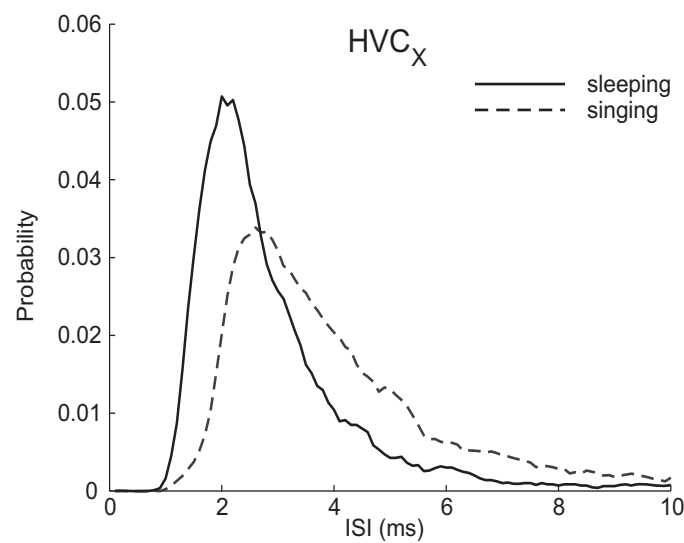**E**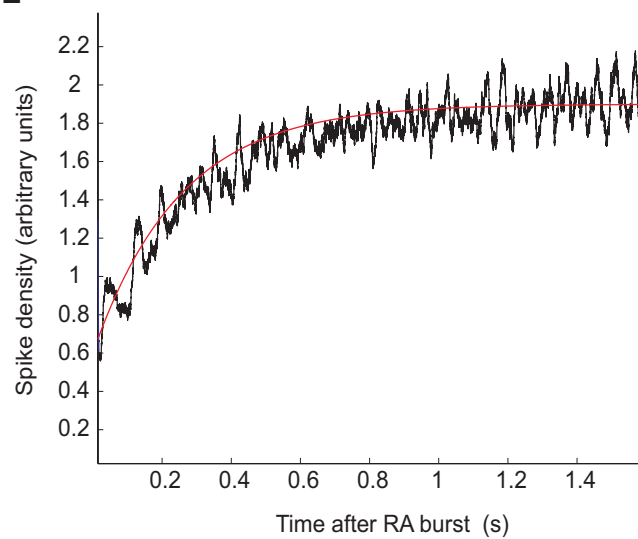**Supplementary Figure 1**

Supplement: Figure S1 — (A–D) Shown are ISI pdfs (normalized to the first 10 ms) measured during singing and during sleep. In all neuron types, sleep-related bursts have lower firing rates, indicated by the rightward shift of ISI peaks. Matching of singing-related and sleep-related ISI pdfs can be achieved by different stretch factors V (see Methods). V = 0.65 for RA neurons in (A), V = 0.9 for HVCI neurons in (B), V = 0.63 for HVCRA neurons in (C), and V = 0.77 for X-projecting HVC neurons (HVCX neurons) in (D). ISI pdfs were produced based on data in [1,10–12]. (E) RA spike histogram for a range of time lags since the last sleep burst, computed for all RA bursts that were followed by a burst-free period of at least 2 s (the histogram is composed of RA single spikes only). The red curve depicts the fit 1.9 − 1.3exp(t/D R), where t is the time lag since the last burst, and D R = 240 ms is our estimation of the RA inhibition time constant. (94 KB PDF) [file pcbi.0030249.sg001.pdf]

**A**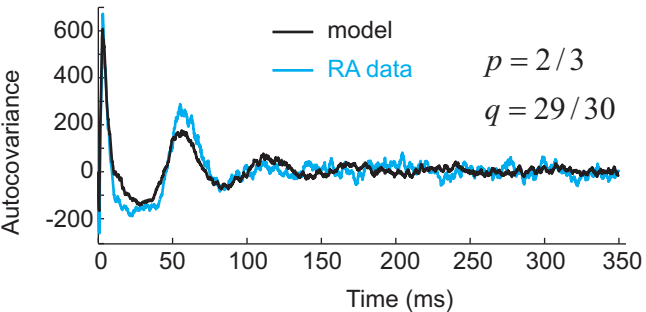**B**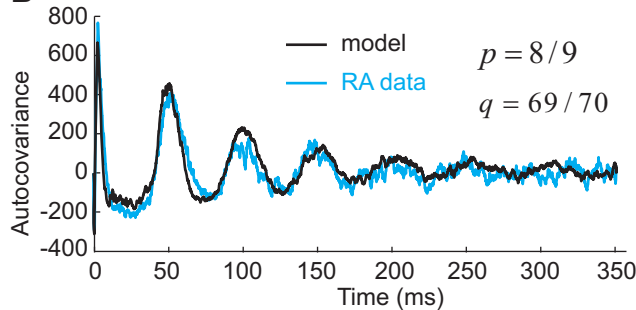

**Supplementary Figure 2**

Supplement: Figure S2 — The autocovariance function C(t) of a spike train ρ(t) (modeled as a sum of delta functions) is a measure of spike density fluctuation and is defined as where is the average firing rate and T is the total duration of the spike train. The characteristic oscillatory behavior of autocovariance functions in RA neurons is well-reproduced by the model. (A) A short survival time of the ground state leads to fast decay of autocovariance oscillations. D R = 240 ms and V R = 0.7. (B) A long survival time of the ground state leads to slow decay of oscillations. D R = 120 ms and V R = 0.67. In (A) and (B), L R = 13 and p R = 0.92. (85 KB PDF) [file pcbi.0030249.sg002.pdf]

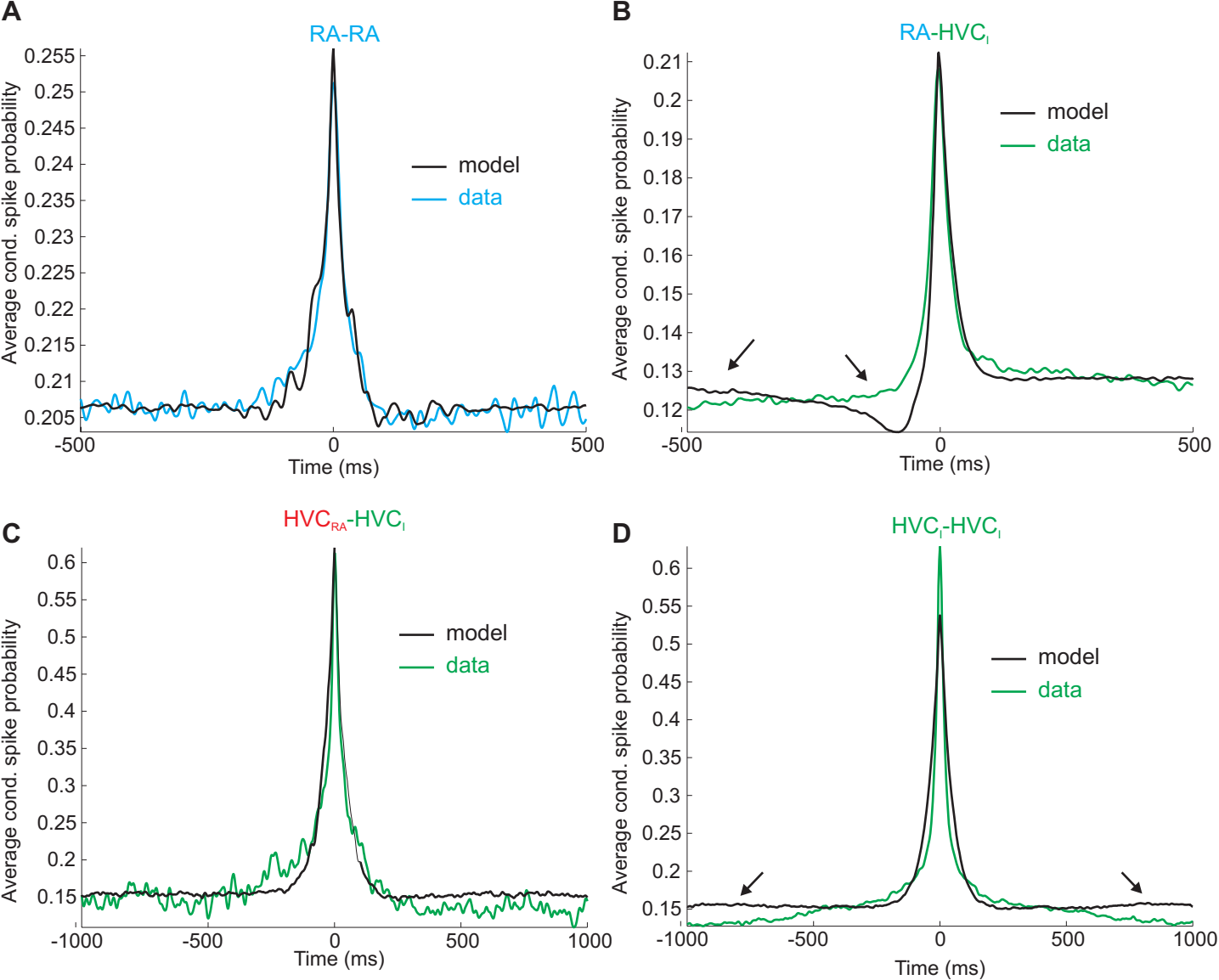

**Supplementary Figure 3**

Supplement: Figure S3 — (A–D) Unlike in Figure 5, no burst epochs (fluctuations in p) were included in the model. Model curves (black) represents the best fits achievable by trial and error. The arrows indicate regions where the quality of fit could not be improved. Same legend as in Figure 5. (49 KB PDF) [file pcbi.0030249.sg003.pdf]
